# Endemic Kaposi’s Sarcoma

**DOI:** 10.3390/cancers15030872

**Published:** 2023-01-31

**Authors:** Perla El Zeinaty, Céleste Lebbé, Julie Delyon

**Affiliations:** 1 Department of Dermato-Oncology, AP-HP Hôpital Saint-Louis, F-75010 Paris, France; 2 INSERM U976, Université Paris Cité, F-75010 Paris, France

**Keywords:** endemic Kaposi sarcoma, Kaposi sarcoma, Kaposi’s sarcoma, KSHV, HHV-8

## Abstract

**Simple Summary:**

Endemic Kaposi’s sarcoma (KS) remains a major public health concern in Eastern and Central Africa, and diagnostic and therapeutic management represents a great challenge in a setting of financial limitations in resource-poor environment. Factors arising from specific ethnic behavior and geographical variation are responsible for the development of KS in the African population infected with HHV-8 mostly through salivary transmission. Our review provides an overview of the clinical characteristics and discusses the therapeutic challenges of this subtype. As endemic KS may follow an aggressive course, it often requires the use of systemic therapy. Immune checkpoint blockade could represent a promising alternative for chemotherapy-refractory endemic KS.

**Abstract:**

Kaposi’s sarcoma (KS) is a common neoplasm in Eastern and central Africa reflecting the spread of *human gammaherpesvirus-*8 (HHV-8), now considered a necessary causal agent for the development of KS. The endemic KS subtype can follow an aggressive clinical course with ulcerative skin lesions with soft tissue invasion or even bone or visceral involvement. In the latter cases, a thorough imaging work-up and better follow-up schedules are warranted. As KS is a chronic disease, the therapeutic goal is to obtain sustainable remission in cutaneous and visceral lesions and a good quality of life. Watchful monitoring may be sufficient in localized cutaneous forms. Potential therapeutic modalities for symptomatic advanced KS include systemic chemotherapies, immunomodulators, immune checkpoint inhibitors, and antiangiogenic drugs.

## 1. Introduction

Kaposi’s sarcoma (KS) is an angioproliferative neoplasm induced by *human gammaherpesvirus-*8 (HHV-8)—*Kaposi sarcoma–associated herpesvirus* (KSHV) targeting the cutaneous and lymphatic systems, with possible involvement of other organs such as the aerodigestive tract [1]. Since its initial description in 1872 by Moritz Kaposi [2], KS has been categorized into four distinct epidemiological and clinical subtypes [3,4]. Classic KS occurring in elderly men of Mediterranean or Eastern European Jewish ancestry, mostly presents with clustered papules and nodules over the extremities with chronic edema. In the 1950s, a more aggressive “endemic form” of KS was reported in immunocompetent young adults and children originating from Sub-Saharan Africa [5]. Iatrogenic KS was initially described in the 1960s, and develops in organ transplant individuals receiving long-term immunosuppressive therapy [6]. Since the 1980s, an epidemic subtype of KS has been described as an AIDS-related disease, developing in individuals seropositive for HIV [7].

Recent discoveries have led to a better understanding of KS viral oncology and its clinical correlation, with a continuous objective to develop promising preventive and therapeutic targets. However, many questions remain to be answered to fully understand and address the multiple features of this disease.

In this comprehensive review, our aim was to focus on endemic KS, and to summarize recent insights into epidemiological and geographical distribution, with a particular emphasis on clinical presentation and specific treatments.

## 2. Epidemiology

### 2.1. Geographical Disparities

The Global Cancer Observatory 2020 data outline the age-standardized incidence rates for KS across continents irrespective of HIV status [8]. With approximately 34,270 new KS cases in 2020 worldwide, the highest rates were recorded in Southern (8.5 per 100,000 in men and 4.7 per 100,000 in women), Eastern (6.0 per 100,000 in men and 2.9 per 100,000 in women), and Central Africa (3.2 per 100,000 in men and 1.1 per 100,000 in women) [8].

While the latest data on KS in Africa reflect both HIV-related and unrelated diseases, it still mirrors to a certain extent the epidemiological pattern that existed before the advent of AIDS. The relative risk of KS in HIV seropositive individuals compared to HIV-seronegative individuals, remains a magnitude lower than would be expected in the United States [9,10,11], highlighting the endemic pattern of KS in Africa with a relatively high proportion of HIV-uninfected cases.

In portions of Uganda, Tanzania, and the Democratic Republic of Congo, incidence rates of endemic KS, approached >9 per 1000 person years [12], whereas North Africa recorded 0.5–1.5 per 1000 person years [12,13]. Hence, the peak prevalence was observed through a narrow belt, extending from the Uganda, Sudan, and Democratic Republic of Congo borders southward through Rwanda and Burundi [14]. This geographical disparity is partially due to known environmental factors such as proximity to volcanic soil [15,16]. Additionally, chronic exposure to iron or aluminosilicate and clay absorption has been hypothesized to induce dermal lymphatic alterations contributing to impaired local immunity and predisposing patients to HHV-8 infection [17].

### 2.2. Age and Gender Distribution

Endemic KS generally occurs at two age peaks: in the pediatric population, among children with a median age varying from 4 to 9 years [18,19,20,21] where the sex ratio is almost one to one, and in the adult population, with a high male preponderance [22,23,24,25]. Presumably, an indirect effect of sex hormones on KS tumorigenesis [26] and a gender difference in immune response might explain the male predominance in KS risk.

### 2.3. Epidemiological Impact of HIV

The epidemiology of KS in Sub-Saharan Africa and South Africa, an area with endemic HHV-8 and high HIV seroprevalence, is driven by both HIV-related KS and endemic KS.

In the context of the HIV epidemic, a major increase occurred in the age-standardized KS incidence in both subtypes (HIV-related and endemic) by approximately 15%, in both female and male adults [27]. Notably, regardless of HIV status, KS became the most common cancer in men and the second most common cancer in women in regions such as Malawi, Zimbabwe, and Eswatini [28,29]. It continues to comprise a substantial percentage of the total cancer burden in Sub-Saharan Africa, with 24% in Mozambique, 27% in Uganda, and 35% in Zimbabwe [29,30,31,32]. Rising numbers of pediatric and adolescent KS were registered in regions of Sub-Saharan Africa [33,34] which have persisted despite the advent of combination antiretroviral therapy [35]. KS subsequently stands as the second most common pediatric malignancy in many Sub-Saharan regions [36,37,38,39].

Since the HIV epidemic, endemic KS has been overshadowed by HIV-related KS and scarce data have been published on HIV-unrelated KS in Africa. Subsequently, high quality and accurate epidemiological data distinguishing between endemic forms of the disease according to HIV status are difficult to obtain.

## 3. Kaposi’s Sarcoma Etiopathogenesis and Other Predisposing Factors

### 3.1. The Etiologic Role of HHV-8

HHV-8 fulfills most of modern-day Koch’s postulates, linking this oncogenic virus to KS [40], as viral replication is known to precede KS tumorigenesis and is mandatory for KS development [41,42,43]. HHV-8 is not ubiquitous, and its prevalence varies greatly across certain regions of the world. The highest rates are reported in Sub-Saharan Africa (50% in Uganda [30,44], 30.7% in Cape Town South Africa [45]) followed by the Mediterranean region with intermediate rates (10% to 30%) [3], whereas general populations in Europe, Asia, and the United States are affected in less than 5% of cases [46]. Importantly, available literature on regional differences in Africa shows HHV-8-seropositivity to be as much as threefold higher in the endemic belt (Uganda) than in African areas from outside the Belt (Zimbabwe and South Africa) [30].

HHV-8 DNA sequences were identified, first in 1994, in all KS lesions from patients with AIDS [1,47]. Initially, HHV-8 infection targeting spindle cells of endothelial origin leads to the production of numerous molecules involved in angiogenesis, such as members of the VEGF–VEGFR family, angiopoietin family, cyclooxygenase 2, and angiogenin [48,49,50], which are key components for HHV-8 oncogenicity. In experimental systems, spindle cells isolated from KS lesions undergo autocrine and paracrine growth effects [3] through several inflammatory cytokines and growth factors including Il-6, oncostatin M [51], scatter factor [52], fibroblast growth Factor 2, and VEGF [53], resulting in the occurrence of KS-like lesions. In vitro, evidence of HHV-8 infection promotes alterations in cellular morphology [54], glucose metabolism [55], growth rate, lifespan, and gene expression [56,57,58,59], leading to a survival proliferative response, but not full neoplastic transformation [3].

The recent focus on signaling pathways shows that HHV-8 immunoregulatory, growth-promoting and oncogenic genes can be expressed without the full execution of the lytic cycle [60,61,62]. Thus, HHV-8 replication may not be required to maintain existing KS lesions, as HHV-8 mostly exists in its latent form in established lesions [63,64].

### 3.2. HHV-8 Salivary Transmission

Long before the outbreak of HIV, KS was seen in children throughout Sub-Saharan Africa, suggesting a non-sexual pathway of transmission [65]. The incidence of HHV-8 infection from birth to 4 years of age was 13.8 per 100 child years [66]. In general, the high incidence of anti-KHSV antibodies in African children [14,67] was linked to salivary transmission of HHV-8 from mother to child as the most likely route of transmission [68,69]. The HHV-8-serologic status of the mother correlated with that of the child [70]; with an increasing proportion of seropositive children in relation to their mothers’ antibody titers [71]. Thirty percent of the children of HHV-8-seropositive mothers had anti-HHV-8 antibodies whereas none of the children of HHV-8-seronegative mothers were seropositive [72]. Additionally, evidence of increasing HHV-8 seroprevalence among children in Uganda from 9% (2 years of age) to 36% (8 years of age) [73], supports a continuous horizontal salivary transmission from child to child in Sub-Saharan Africa [74,75]. This pattern may result from common behavioral practices in endemic regions such as applying saliva to children’s bite sites to relieve itching, kissing, sharing of toothbrushes, premastication of food for infants, and candy sharing [14,76].

### 3.3. Infectious Agents

Although HHV-8 infection is necessary for the development of KS lesions, it is not sufficient, and several factors could contribute to the maintenance of the disease [3].

Environmental factors such as concomitant infections with parasitic agents may explain part of the geographical variation in HHV-8 prevalence [77]. According to several case–control studies conducted in Uganda, a high proportion of KS patients were bitten by bloodsucking arthropods, suggesting that they may contribute to the seroconversion to HHV-8-positivity and ultimately to the pathogenesis of KS [78]. Although these co-infections were associated with an increase in HHV-8 seropositivity [77], their direct effect on saliva shedding was not confirmed as they may result from common practices of saliva sharing in endemic regions [76]. Furthermore, dysregulation in the immune response caused by iterative malaria infections, may somehow be a co-factor in HHV-8 replication and/or KS spindle cell proliferation [14].

### 3.4. Socioeconomic Status

Since infection with HHV-8 has been consistently associated with KS lesions, several reports have examined the association between HHV-8 seroprevalence and low socioeconomic status markers in South Africa particularly among HIV negative individuals [65,79]. The risk of acquiring HHV-8 infection increased in individuals with specific behavioral and sociodemographic characteristics, such as paid domestic work, unemployment or history of undernutrition during childhood manifesting in delayed age at menarche for women [79,80,81]. Similarly, a high level of education reflects a more favorable socioeconomic status, suggesting slower transmission of HHV-8 through low exposure to the virus [67,72,79].

## 4. Diagnosis

Distinguishing KS cutaneous lesions from other benign or infectious vasoproliferative tumors involving the skin can be challenging. Bacillary angiomatosis illustrates one of the clinical misdiagnoses in the African region exhibiting both morphological and histological similarities [82]. When resources permit, skin biopsy for routine histological examination combined with histochemical and immunohistochemical stains remains the preferred diagnostic tool.

The patch stage presents with patchy dilated leaky vessels mostly in the reticular dermis accompanied by a perivascular inflammatory infiltrate composed of lymphocytes and plasma cells. Plaque stage KS is characterized by diffuse dermal proliferation of spindle cells arranged in fascicles separating collagen bundles and forming irregular jagged-outlined vascular spaces. There is commonly an extravasation of red blood cells and hemosiderin deposits. Nodular lesions consist of a dense well-defined infiltrate of spindle cells along replacing the dermal collagen and forming pseudo-vascular spaces filled with red blood cells [4,24,83]. Immunohistochemistry using a monoclonal antibody targeting LANA-1 (Latent Nuclear Antigen) contributes to the identification of HHV-8 within KS cells in KS lesions [4,84]. It is a very helpful diagnostic tool in addition to routine HE staining.

Cellular components of KS lesions typically involve spindle cells expressing endothelial markers such as CD34 and CD31 of vascular origin, as well as VEGF receptor 3, LYVE1, and podoplanin, highlighting its lymphatic endothelial cell origin [4,24]. Recent immunohistochemical data showed that spindle cells also express vimentin, indicating a potential mesenchymal origin; HHV-8 infection may trigger the mesenchymal-to-endothelial transition contributing to the development of KS [24,85,86]. To date, the immunostaining technique is unavailable in most centers in Africa [84].

## 5. Prognosis, Staging, and Workup

Four clinically distinct categories of endemic KS have been identified but no staging classification has been validated to date: (1) a benign nodular form mimicking classic KS mostly occurring in young adults; (2) an aggressive form with local invasion of underlying soft tissue and bone; (3) a disseminated disease including florid mucocutaneous and visceral involvement, similar to HIV-associated KS before the advent of highly active antiretroviral therapy (HAART); and (4) a fulminant lymphadenopathic form with rapid lymph node and visceral disease particularly seen in children [5,75]. Patients should undergo a thorough clinical examination followed by a comprehensive blood test including complete blood count, basic and metabolic panel, and HIV serology completed with DNA or RNA PCR when the diagnosis is questionable [83]. The use of total body imaging, bronchoscopy and gastrointestinal endoscopy for the screening of visceral KS, and bronchial and digestive mucosal KS are tailored according to patient’s symptoms [87]. This baseline work-up, customized according to available diagnostic tools, allows clinicians to address patients following three prognostic stages: localized nonaggressive, locally aggressive, and disseminated KS [83,88,89]. Based on their clinical practice, experts recommend regular clinical follow-up for patients with KS, combined with standard blood tests including complete blood count, and renal and hepatic function panels. Only when visceral localization is suspected upon specific signs or symptoms, are supplementary imaging and endoscopy needed.

## 6. Endemic Kaposi’s Sarcoma Presentation and Variants

### 6.1. Clinical Features

Endemic KS occurs in young adults living in Sub-Saharan Africa, and typically manifests as localized mucocutaneous disease [90,91]. It presents with cutaneous lesions clustered over the lower-limbs mimicking classic KS [23], potentially causing an itching or burning sensation [16].

**Figure 1:**
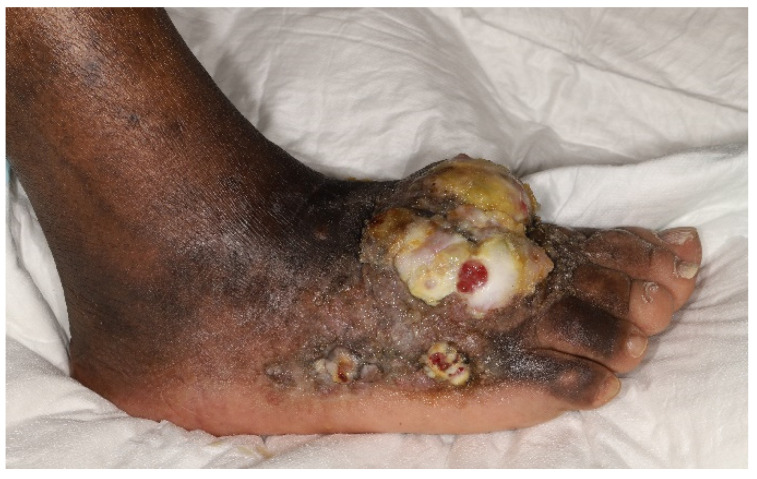
Clinical photograph of a 62-year-old immunocompetent man of African origin diagnosed with endemic Kaposi sarcoma. Note the well demarcated lobular central mass with surrounding nodular areas peripherally over the dorsum and the medial aspect of the right foot with accompanying lymphoedema of the entire lower limb. (Dermatology Department, AP-HP Hopital Saint-Louis—June 2019).

As the cutaneous disease progresses, skin lesions may transition through the patch, plaque, nodular, infiltrative, or florid forms [90]. Nodular KS is an indolent subtype limited to skin involvement. Florid KS refers to exophytic, locally aggressive and infiltrative lesions that extend deeply into subjacent musculoskeletal structures [92]. (Figure 1) In a recent study, bone involvement (31%) was the most frequently extracutaneous involved site identified on FDG PET/CT [93]. Such “anaplastic forms”, referring to high local aggressiveness, are rarely inaugural [94] and mostly result from a longstanding cutaneous disease [19,36]. As lymphoedema of the lower limbs represents approximately 17% of cutaneous manifestations [95], it remains a frequent complication of endemic KS and can be difficult to manage [96]. Chronic lymphatic stasis [97,98] seems to result in a continuous angiogenic stimulus secondary to the locoregional immunological response and in the subsequent development of collateral lymphatic and endothelial circulation [94].

Endemic KS may less frequently involve extracutaneous sites including lymph nodes and visceral organs, particularly the respiratory and gastrointestinal tracts [90]. In an observational retrospective cross-sectional study conducted in South Africa [99], only 1.4% of KS patients were diagnosed with visceral KS predominantly within the gastrointestinal tract. The limited cases of identified visceral disease may be imputed to underdiagnosis, as concomitant mucocutaneous lesions are more readily accessible and biopsied [99]. Considering limitations in resources where diagnostic tools such as bronchoscopy and endoscopy are generally lacking, alternative clinical assessments of respiratory and digestive tract involvement were proposed [19]. Standard chest X-rays may contribute to diagnostic staging. Pulmonary KS may present with reticulonodular infiltrate or pleural effusions, refractory to empiric anti-tuberculosis or antibiotic treatment [19]. Abdominal visceral disease is suspected in the presence of ascites or bloody stool, while dysphagia improving with chemotherapy initiation is suggestive of an upper gastrointestinal disease [19].

### 6.2. Pediatric Variant

Endemic KS in children presents with clinical features different from those described in the adult subtype [24,100,101,102]. It is mostly characterized by lymph node involvement (ranging from 50–60% of cohorts) and woody edema (ranging from 40–70% of cohorts) [20,21]. Diffuse lymphadenopathy occurs in approximately one fourth of pediatric cases in Sub-Saharan Africa, even in the absence of prototypical cutaneous lesions or woody edema which represents a major diagnostic challenge among African regions [19,20,36]. Interestingly, the lymphadenopathic KS variant has not been observed outside the Sub-Saharan Africa region, and remains the most common and unique clinical manifestation of endemic pediatric KS. In the 40 cases reviewed by Dutz and Stout [25], multifocal lymphadenopathy was described in 44.4% of African children while only 4–5% of non-Africans presented with similar features.

A distinct pattern of clinical presentation between HIV-negative endemic KS and HIV-related KS in the African setting was established by El-Mallawani et al. when comparing characteristics from both cohorts in the pediatric population of Malawi [20]. Such distinctive features highlighted the uncommon occurrence of oral lesions as well as lower visceral involvement in the HIV-negative endemic subtype. Only 10% of oral cavity involvement was reported among HIV-seronegative children with endemic KS, with over 60% having cytopenia (i.e., anemia) as the main laboratory abnormality [20,21].

The modified “Lilongwe Pediatric KS Staging Classification” [103], which has yet to be validated, includes four specific groups that are significantly correlated with prognostic outcomes [19,103]. Children with mild to moderate cutaneous and/or oral involvement (stage 1: 1–9 lesions (1A); 10–19 lesions (1B)) and those with lymphadenopathic disease (stage 2) exhibit good prognostic outcomes with high percentages of survival of 100% and 76%, respectively [19,104]. Patients with woody edema (stage 3: <10% (3A) or >10% (3B) of body surface area) are associated with a more chronic disease course [19], demonstrating an approximately 78% survival rate [104]. KS presenting with visceral involvement and/or disseminated lesions (≥20 lesions) categorized into stage 4 [19] demonstrates lower yet overall positive outcome despite significant resource limitations with a two-year overall survival estimate of more than 50% [103,104,105].

### 6.3. Musculoskeletal Skeletal Involvement

In his initial description of KS, Moritz Kaposi reported cases with deep ulcerating lesions resulting in underlying periosteal damage and osteolytic lesions [106], referring to skeletal involvement by KS. Similar bone lesions are more frequently reported in the African population than in any other epidemiological subtypes [107,108].

Patients with bone involvement of KS may have localized pain [107], while others present with asymptomatic diffuse osteolysis [109]. Some rare cases of neurologic symptoms secondary to spinal cord compression have been reported [110].

A review of 66 cases of KS with skeletal involvement conducted by Caponetti et al. [107] showed that almost all patients with endemic KS had extraosseous disease. Skin lesions were contiguous to subjacent osseous disease, forming diffuse infiltrative or painful ulcerating florid cutaneous tumors, eroding deep down into the underlying periosteum. In the endemic form of KS, there is a propensity for involvement of the extremities, as seen in our case (Figure 1) [106,111].

**Figure 2:**
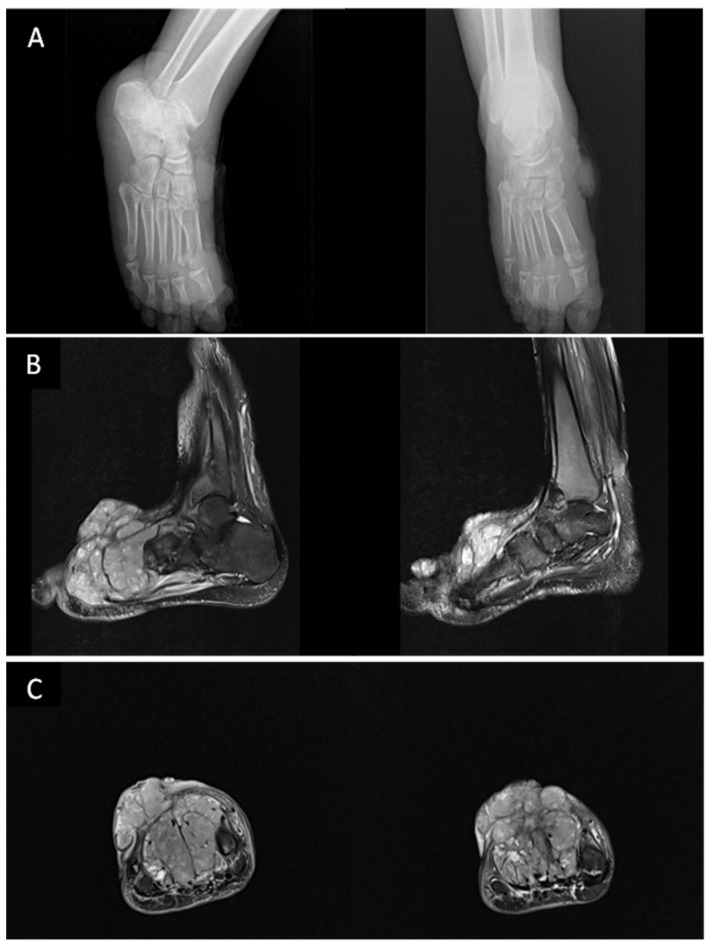
Imaging of the patient described in Figure 1. (**A**) Plain radiograph in the anteroposterior and lateral projection of the right foot showing lobulated soft tissue swelling involving the anterior and medial malleolus aspect without any associated periosteal reaction of the adjacent bones. (November 2015). (**B**,**C**) Magnetic resonance imaging (MRI) (Sagittal T2 FS images (**B**) Coronal T2 FS (**C**)) shows multiple enhanced exophytic cutaneous and subcutaneous masses of the right foot with osseous involvement of underlying structures (the main lesion overlying the second, third, and fourth metatarsal bones, lobulated dorsal mass adjacent to the middle phalange of the second toe) associated with a local invasion of the extensor tendons and close contact to flexor tendons (February 2019).

Most bone lesions are osteolytic with cortical erosion or destruction secondary to KS lesions arising in the cortex or the periosteum. The conventional radiographic findings are variable, such as uniform rarefaction, cystic lesions [107], or bubbly appearance [112]. As it may also result from external cortical pressure from overlying cutaneous florid tumors, KS-related osteolytic lesions are infrequently associated with periosteal reaction with better characterization of hypodense lytic changes on CT imaging [112,113]. Trabecular bone involvement also appears as hypoattenuating bone lesions on CT imaging. Whenever contiguous bone involvement is suspected, MR imaging demonstrates enhancement of soft tissue and is more accurate for evaluating subcutaneous extension and for detecting spongy bone and diffuse bone marrow involvement [114,115,116,117]. Although bone scan findings may be normal, it is particularly helpful in detecting asymptomatic areas of bone lesions showing irregular radionucleotide uptake [112,116,118] (Figure 2).

In patients with aggressive localized infiltrative tumors, widespread disease, or bone pain, a thorough evaluation is warranted for detecting bone lesions. The detailed work-up should include CT scans for detecting cortical lytic changes and MR imaging for better identification of soft tissue masses and spongy bone involvement. Compared to conventional imaging studies, FDG-PET is mostly beneficial for whole-body staging and is also helpful for distinguishing visceral or skeletal involvement which can herald a worse prognosis [119]. A retrospective study including 75 patients with KS of all four types [93], points to a major efficacy of FDG-PET in evaluating KS cutaneous and visceral involvement, with more than 85% sensitivity and specificity for musculoskeletal lesions.

### 6.4. Diagnostic Challenges in Resource-Limited Settings

In low- and middle-income (LMI) settings, conventional radiography is readily available to help detect bone erosion or frank destruction as well as periosteal reaction. In cases where bone lesions go unrecognized and are difficult to detect on X-Ray, the use of a CT scan may provide better identification of osteolytic changes with cortical bone involvement [91,114,116,117]. On another note, South Africa stands among the minority of countries providing PET scanners in both FDG and non-FDG tracers. However, considering an elevated financial cost in the public sector and the limited equipment, the use of PET/CT is limited in sarcomas for staging purposes for selected cases, for diagnosis and surveillance purposes, and in cases of suspected recurrences [120].

## 7. Therapeutic Management

### 7.1. Treatment Strategy for Adult Form: European Standards

Therapeutic management for patients with endemic KS must be tailored to the number of lesions, the extent of the disease and its functional consequences, and the presence of visceral involvement. (Table 1)

Benajiba et al. [121]. examined the types of treatment given to patients with endemic or classical KS, in a large monocentric retrospective study and showed that local treatment was the most frequently used (45%), followed by systemic therapies (41%, including doxorubicin, taxanes, and interferon), and therapeutic abstinence (14%). Watchful monitoring is appropriate for asymptomatic patients in the absence of aggravating factors in a slow-progressing disease.

Minimally aggressive and localized forms of KS can respond favorably to local therapy but often tend to recur [122]. The main local therapeutic options include cryotherapy, surgical excision, intralesional chemotherapy using vincristine [83,123,124], or localized radiotherapy for painful lesions of the lower extremities [4,83,125].

In widespread, progressive, and symptomatic forms, or with visceral involvement, systemic treatment is generally warranted [126]. Based on a French cohort, the endemic KS subtype was significantly associated with systemic treatment initiation [121]. The preferred chemotherapy regimens were pegylated liposomal doxorubicin (PLD) (20 mg/m^2^ intravenous perfusion every three weeks) and alternatively paclitaxel (PTX) (80 mg/m^2^ weekly, continuously, or three weeks on and one week off, being the preferred schedule for non-HIV KS) [83,124,127]. Pegylated-liposomal encapsulation has been proven to enhance the therapeutic effect of doxorubicin used in the ABV combination (adriamycin-vincristine-bleomycine) showing superior response rates of 45.9% compared to only 24.8% for the ABV regimen [128]. Due to their potent cytotoxic and antiangiogenic activity [129,130,131], PLD and PTX have shown great clinical efficacy in HIV-associated KS, achieving response rates in the range of 50 to 60% [132]. Similar studies focusing on the efficacy profile of PLD and PTX in HIV-negative forms of KS demonstrated major clinical responses while exhibiting good tolerability to treatment [133,134,135].

Alternatively, immune-modulating agents such as low-dose interferon alpha and its pegylated derivatives, used at three to five million units, represent an effective and well tolerated therapeutic modality for endemic KS in younger patients with normal cardiac function [136,137].

Recent preliminary data suggest the efficacy of anti-PD-1 in patients with classic and endemic KS. Major clinical and metabolic partial responses were obtained following nivolumab in two severe endemic KS patients with cutaneous, lymph node, muscular, and bone extension [138]. A multicenter phase II trial [139] demonstrated that treatment with anti-PD1 showed efficacy for patients with classic and endemic KS with cutaneous extension requiring systemic treatment, with the best overall response rate above 70%.

In an open-label single-arm phase I/II study [140], pomalidomide, a thalidomide analog, was used in 28 individuals with advanced cutaneous KS. When administered at 5 mg orally per day for 21 days of a 28-day cycle, pomalidomide was proven safe and active against KS, in both participants with or without HIV, with an overall response rate of 71% and a median progression free survival of 10.2 months. Through VEGF inhibition, pomalidomide exhibits antiangiogenic capacity and constitutes a promising therapeutic class that has been recently approved by the FDA for use in adult patients with HIV-related KS (after the failure of highly active antiretroviral therapy) and non-HIV-related KS [141]. There are currently no published data regarding the efficacy and safety of thalidomide analogs in individuals with endemic subtypes. However, an ongoing larger clinical trial aims to analyze the efficacy of pomalidomide for HIV-associated KS in Sub-Saharan Africa (NCT03601806).

While endemic KS patients usually respond favorably to systemic chemotherapy and/or radiation therapy, some patients may be refractory to these treatments. In cases of painful ulcerating rapid-growing KS tumors, with bone and/or muscular extension, and failure of standard and experimental treatments, limited amputation may be necessary [110].

### 7.2. Therapeutic Considerations in Resource-Limited Settings

In 2020, there were an estimated 25,010 new cases of KS on the African continent and 13,066 deaths in the region [8]. The global cancer burden is growing in Sub-Saharan Africa and deaths remain high due to limited access to tools for early diagnosis and to the challenging provision of anticancer treatments in resource-poor settings [142,143]. While PLD and PTX have been proven highly effective in the treatment of endemic variants of KS [132], optimal chemotherapy regimens have not been systematically evaluated in LMI countries. In such settings, bleomycin and vincristine are commonly used due to their lower cost and wider availability, and orally administered etoposide is readily incorporated in an outpatient setting [144]. In this view, a prospective randomized control study [145] was conducted to assess the noninferiority of bleomycin and vinblastine or etoposide in combination with HAART compared with paclitaxel plus HAART. The results interpreted in HIV-related KS and showing greatly superior outcomes with PTX plus HAART could be extrapolated to endemic KS in African areas.

### 7.3. Treatment Strategy for Pediatric Forms

Given the rarity of pediatric KS, there are no therapeutic guidelines for pediatric KS. Most therapeutic modality options are extrapolated from published articles about KS in adults [131].

Chemotherapy remains the cornerstone for treating children with endemic KS [22,146]. The combination regimen of bleomycin and vincristine is now considered the standard chemotherapeutic regimen for pediatric KS in Lilongwe, Malawi, and Tanzania [147]. It represents an alternative to the originally described vincristine monotherapy and offers a practical option for outpatient approaches in resource-limited settings [103]. However, chemotherapy is not routinely available in certain LMI regions with uneven access to adequate cancer care contributing to high mortality rates in KS refractory cases [148].

The risk-stratification platform based on the “Lilongwe Classification” seeks to define therapeutic options predicting which patients may have a favorable prognosis with bleomycin and vincristine (BV) and those who will eventually require alternative chemotherapy [75,149]. All patients with stage 1B and above are initially offered four cycles of BV regimen. Stage 4 patients and those who failed to respond to the BV induction phase require an intensified chemotherapy regimen composed of eight cycles of doxorubicin, bleomycin, and vincristine (ABV) [103]. In cases of treatment failure, KS relapse or as an initial treatment in circumstances where ABV is contraindicated or according to the clinician’s preferences, paclitaxel, which became available in 2013, is given for at least six cycles [101,150]. In a recent pediatric cohort [150], 62.5% of stage 4 patients who failed to respond to previous chemotherapy regimen were able to achieve complete clinical remission with paclitaxel thus mirroring previous earlier experiences in Mozambique displaying 60–90% partial clinical response [101].

### 7.4. Current and Future Perspectives

The World Health Organization (WHO) developed a Model List of Essential Medicines (EML) encouraging countries to prioritize specific anticancer drugs in national essential medicine lists and national reimbursable lists [151]. The list established in 2021 addresses KS treatment indications with bleomycin, vinblastine, doxorubicin, and paclitaxel [152].

Furthermore, numerous public–private collaborations seek to supply and provide the quality cytotoxic medications required in treatment centers across Sub-Saharan Africa at affordable prices. Paclitaxel and doxorubicin appear among drugs prioritized for KS guidelines in countries such as Tanzania, Uganda, Zimbabwe, and Malawi [153].

## 8. Conclusions

KS is one of the most common neoplasms in Sub-Saharan Africa and parallels the endemicity of HHV-8, the causative oncogenic organism. Other etiologic factors are specifically related to ethnic practices in African populations, such as HHV-8 salivary transmission.

Endemic KS commonly presents with a monomelic variant consisting of a lymphedematous limb with large florid tumors locally infiltrating subjacent soft tissues and bones.

However, although endemic KS is a leading malignancy in Sub-Saharan Africa, the diagnosis is challenging in resource-limited environments, as immunostaining techniques are not always available.

KS remains a major public health concern in endemic regions as financial limitations represent a great challenge for conducting large therapeutic studies aiming to improve specific therapeutic management. To date, there are no curative therapeutic options, as HHV-8 is not eradicable. The purpose of tailored management is to adapt therapy to patient’s fitness and quality of life requirements. Treatment guidelines designating PLD and PTX as preferred first-line systemic agents are however, infrequently used because of their low availability and high cost. Further prospective trials are required to validate the use of PD1 blockade or pomalidomide in patients with endemic KS.

## Figures and Tables

**Table 1:** Therapeutic options for Kaposi sarcoma in terms of patients’ clinical presentation.

Clinical Presentation	Therapeutic Strategy
**Adult Kaposi Sarcoma** Indolent asymptomatic slow progressing disease Localized cutaneous/oral disease	Therapeutic abstinence or Local therapies: - Surgical excision - Radiotherapy - Local or intralesional chemotherapy - Topical immune modifying agents (Imiquimod) - Cryosurgery and CO_2_-Laser
**Adult Kaposi Sarcoma** Advanced rapidly progressive disease Disseminated cutaneous/oral disease Lymph node disease Visceral localization Soft tissue extension +/− severe lymphedema +/− painful lesions	Systemic chemotherapy - Pegylated liposomal doxorubicin (20 mg/m^2^ IV every 3 weeks) - Paclitaxel (80 mg/m^2^ IV weekly, 3 weeks on 1 week off) - Vinblastine (3 mg/m^2^ IV weekly) - Bleomycine (5 mg/day IV for 3 days every 2 weeks) - Etoposide (100 mg/day orally 1–3 up to 1–5 every 3 weeks) Immune-modulating agents - Interferon alfa (3 million units 5 times a week for 2 weeks then 2–6 million units 3–6 times a week) - Peg-interferon-α2a (180 micrograms/week SC) Immune checkpoint inhibitors (in selected cases) - Nivolumab (3 mg/kg IV every 2 weeks) - Pembrolizumab (200 mg IV every 3 weeks) Anti-angiogenic therapies - Pomalidomide (5 mg orally per day for 21 days of a 28-day cycle) +/− Local therapies (radiotherapy or surgical resection) Common Dosing Adjustments for Systemic Chemotherapy in LMIC settings - Paclitaxel (100 mg/m^2^ IV every 3 weeks) - Vinblastine (2 mg IV every 3 weeks) - Bleomycine (15 units/m^2^ IV every 3 weeks) - Etoposide (50 mg up to 100mg/BID orally on days 1–7 of each 21-day cycle)
**Pediatric K** **aposi Sarcoma**	BV regimen (4–6 cycles at two-week intervals for induction treatment then additional 4–6 cycles at monthly basis for consolidation treatment) +/− HAART - Bleomycin 15 units (U)/m^2^ IV - Vincristine 1.4 mg/m^2^ IV ABV intensified regimen (6–8 cycles at three-week intervals) +/− HAART - Bleomycin 10–15 units (U)/m^2^ IV - Vincristine 1.4 mg/m^2^ IV - Doxorubicin 25 mg/m^2^ IV Paclitaxel (6 cycles at four-week intervals) - Paclitaxel 100–135 mg/m^2^ IV - Premedication (dexamethasone, H-1, and H-2 receptor antagonist)
